# The National Adult Inpatient Survey conducted in the English National Health Service from 2002 to 2009: how have the data been used and what do we know as a result?

**DOI:** 10.1186/1472-6963-12-71

**Published:** 2012-03-21

**Authors:** Anna DeCourcy, Elizabeth West, David Barron

**Affiliations:** 1 School of Health and Social Care, University of Greenwich, London, UK; 2 Reader in Organisational Sociology, Said Business School, University of Oxford, Oxford, UK

## Abstract

**Background:**

When it was initiated in 2001, England's national patient survey programme was one of the first in the world and has now been widely emulated in other healthcare systems. The aim of the survey programme was to make the National Health Service (NHS) more "patient centred" and more responsive to patient feedback. The national inpatient survey has now been running in England annually since 2002 gathering data from over 600,000 patients. The aim of this study is to investigate how the data have been used and to summarise what has been learned about patients' evaluation of care as a result.

**Methods:**

Two independent researchers systematically gathered all research that included analyses of the English national adult inpatient survey data. Journals, databases and relevant websites were searched. Publications prior to 2002 were excluded. Articles were also identified following consultation with experts. All documents were then critically appraised by two co-authors both of whom have a background in statistical analysis.

**Results:**

We found that the majority of the studies identified were reports produced by organisations contracted to gather the data or co-ordinate the data collection and used mainly descriptive statistics. A few articles used the survey data for evidence based reporting or linked the survey to other healthcare data. The patient's socio-demographic characteristics appeared to influence their evaluation of their care but characteristics of the workforce and the. At a national level, the results of the survey have been remarkably stable over time. Only in those areas where there have been co-ordinated government-led campaigns, targets and incentives, have improvements been shown. The main findings of the review are that while the survey data have been used for different purposes they seem to have incited little academic interest.

**Conclusions:**

The national inpatient survey has been a useful resource for many authors and organisations but the full potential inherent in this large, longitudinal publicly available dataset about patients' experiences has not as yet been fully exploited.

This review suggests that the presence of survey results alone is not enough to improve patients' experiences and further research is required to understand whether and how the survey can be best used to improve standards of care in the NHS.

## Background

National patient surveys were first proposed in England in a Government policy document *The New NHS: Modern, Dependable *[1]. The survey programme was intended to provide "systematic evidence to enable the health service to measure itself against the aspirations and experience of its users, to compare performance across the country, and to look at trends over time." Further reference was made to improving patient involvement in *The NHS Plan *[2], which mentioned "patients' surveys and forums to help services become more patient-centred".

The first survey of adults who had been treated as an inpatient in an Acute Hospital in England was conducted in 2001/02 and surveys have been repeated almost annually since. The survey programme has generated six consecutive years of data, freely available through the Economic and Social Data Service (ESDS).

Government policy documents continue to emphasise the importance of a more 'patient-centred' NHS in, for example, *Equity and Excellence *[3] and *Liberating the NHS: Legislative framework and next steps *[4]. The *NHS Outcomes Framework 2011/2012 *[5] includes patient experience of care as one of five core domains to be targeted in the NHS in 2011/12. These are:

1. Preventing people from dying prematurely,

2. Enhancing quality of life for people with long-term conditions,

3. Helping people to recover from episodes of ill health or following injury,

4. Ensuring that people have a positive experience of care, and

5. Treating and caring for people in a safe environment and protecting them from avoidable harm.

With patient-centred care high on the political agenda, and media reports of contradictions between the survey and acute hospital trust inspection results [6], it is vital to identify where and how data gathered by this survey programme are being used and to what effect.

This study asks how national adult inpatient survey data have been used in research to date to facilitate patient-driven improvements in acute trusts in England. We also set out to summarise the main findings of the programme so far and to identify the potential for further research. A preliminary search found no existing reviews of research using national adult inpatient survey data; this paper is intended to fill that gap. Our research questions are:

1. To what extent has data gathered by the national inpatient survey programme been used as the basis of empirical analyses and how might the quality of these outputs be described?

2. What are the main conclusions about patients' experiences of care in acute hospitals in the NHS that can be drawn from the accumulated data obtained by the survey programme?

### Questionnaire and Survey Background

There are numerous patient experience surveys conducted annually in Europe, the USA and Canada [7]; Delnoij (2009) states that these endeavour to obtain "detailed reports of what actually happened to patients during a hospital stay" not just an overall measure of satisfaction. In England, the adult inpatient survey seeks to collect the views of recent patients about "how good the hospitals are and how they can be improved" [8] as well as some socio-demographic information about each respondent.

The two main current uses of the inpatient survey are to aid the regulatory functions of the Care Quality Commission (CQC) and to assist local improvement. The patient survey comprises over 20 per cent of the items in the CQC's Quality and Risk Profiles (QRP) [9], which assess compliance with Essential Standards of Care. It is unclear to what extent acute NHS trusts use data locally; there appears to be no systematic collection of information about how these data are used within hospital Trusts.

Responsibility for the programme began with the Department of Health (DoH) and transferred to the Commission for Health Improvement (CHI) in 2003. In April 2004 authority passed to the Healthcare Commission and from October 2008 the survey has been overseen by the CQC. Official results and key findings have been reported annually, with trust level reports made available from inception [10].

The survey was designed by and is conducted through the Picker Institute Europe on behalf of the regulatory body. Questions for the survey were developed through pilot studies that sought to understand the issues that are of importance to patients [11].

The survey questions are organised under thirteen headings that cover the journey of the inpatient from arrival at the hospital to discharge. Most ask respondents to select one option from a set of pre-defined responses. Patients may also include their own comments in a section at the end.

Using detailed written guidance [12,13], the surveys are conducted according to a standard protocol by an approved contractor on the trust's behalf or, in a small proportion, by the trust itself. For each trust, the sample consists of 850 consecutively discharged patients aged 16 and over. Questionnaires are posted to selected patients, and up to two reminders are sent to non-responders at 2-weekly intervals. For responders who are unable to read English, a free telephone line is provided that aims to connect responders to a translator who would complete the questionnaire on their behalf over the telephone.

The highest response rate (64 per cent) was achieved in 2002 [14]. Response rates have declined over the years to 50 per cent in 2010 [15].

## Methods

A preliminary search for systematic reviews that made use of national inpatient survey data was conducted using the Campbell Collaboration, Evidence for Policy and Practice Information (EPPI) and Co-ordinating Centre, Database of Abstracts of Reviews (DARE) and Trip Databases. The sites were interrogated using variations of the term 'systematic review' AND 'inpatient survey' in July 2010 and October 2010. The searches indicated that no review had been conducted.

### Search strategy

Websites were selected by their relevance to the NHS, the national inpatient survey, following consultation with experts in the field [*see Acknowledgements*] and websites of organisations approved by the CQC to conduct the inpatient survey. Journals and databases were interrogated and a literature search completed by the Royal College of Nursing (RCN). For details of the search strategy and the RCN outcome, please see Additional file 1.

Reference manager packages such as End Note and Mendeley were tested for suitability for managing the research collection process. Mendeley [16] was selected as the authors found that it had some advantages:

a. The desktop version is free.

b. The interface between the on-line and desktop versions is very easy to use and allows automatic extraction of article details.

c. The identified research (PDFs, word docs etc.) can be attached for ease of review.

d. It allows notes to be put on documents (both inside the document itself, and as part of the document detail, i.e. author, title, year), again to assist the review process and include reminders of justifications for article exclusions/inclusions.

e. It is easy to share among co-authors.

A scoping search was conducted in July 2010, followed by a full systematic investigation conducted by two independent researchers in October. The search was also updated by one researcher in January 2011. Search results were screened by the researchers based on title and date of publication and if these suggested that the paper fit the inclusion criteria, the abstract was read. Full papers were obtained for all unique research articles identified in the search as well as a group of papers that were recommended by experts in the area. These were read by the two reviewers who each formed a view as to whether they met the inclusion criteria. Where they disagreed, a third reader was consulted. Papers were read in full and further exclusions made.

### Search terms

To ensure a consistent and replicable approach, PICO (population, intervention, comparison and outcome) methodology was adopted to create an extensive list of search terms, highlighting differences in phraseology, spelling and acronyms. The search terms were combined with lessons learnt from performing the preliminary search and adapted according to search engine capability. For a breakdown of search terms and mechanisms used to identify papers for review, please see Additional file 2.

### Eligibility criteria

Identified papers made reference to a minimum of one year of national inpatient survey data. Papers reporting response rates only were excluded. Research comprising direct analysis of raw figures, or extracts from annual regulator-led findings, were both included. Articles reporting results at trust level that included national comparisons were accepted. Papers reporting results for one trust only, with no national referencing, were excluded. Research that made use of models which used the inpatient survey as one of numerous indicators, such as the star ratings system or DoH toolkit for analysts [17], were excluded as the inpatient survey was not the main focus. Papers that discussed the survey in general without including or analysing data were excluded, as were articles based on another paper without reporting independent analyses.

No exclusions were applied regarding ethnicity, gender, language [18] or country of origin. However, the search was limited to publications no earlier than January 2002 because the first survey was conducted in 2001/02. Research based solely on another survey, such as United States' National Inpatient Survey (NIS) or Patient Experiences Questionnaire, were excluded. Grey literature was beyond the scope of the search and consequently not included.

### Controlling for bias

To ensure the search was comprehensive and to reduce the impact of researcher selection bias, two research fellows systematically and independently conducted searches of the literature and their findings were amalgamated. A third reader assisted with the process of deciding which papers should be included. Search terms, sources and eligibility criteria were pre-agreed to ensure consistency and make certain the requirements were fully understood. A wide variety of research studies were included and experts were consulted [*see Acknowledgements*], to ensure a fair and wide search.

## Results

**Figure 1:**
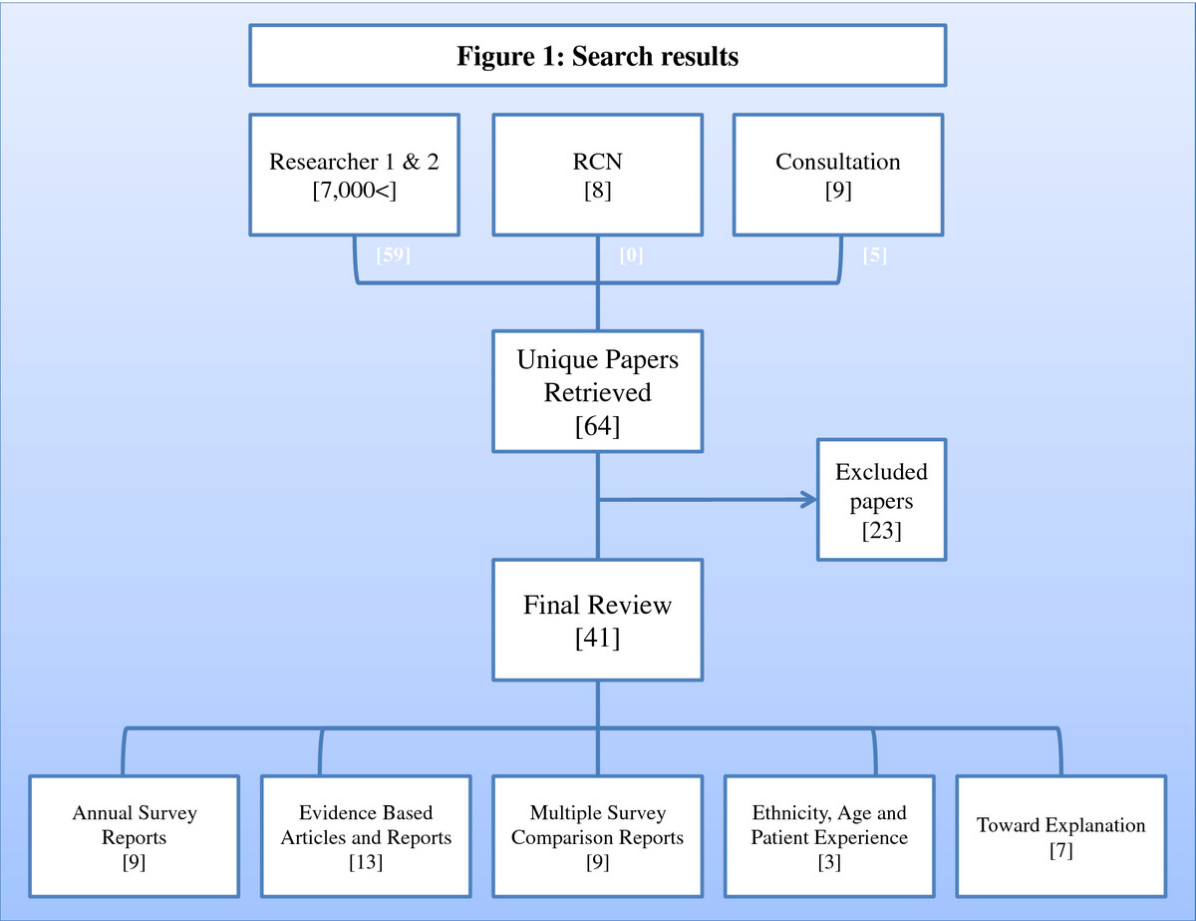
**Search results**.

Identified papers were amalgamated and duplicates removed, as outlined in Figure 1.

64 papers were selected as meeting initial review criteria. 23 were later removed following screening of whole documents. The 41 papers included for final review were broken down into the following five categories:

• Annual survey reports [9]

• Evidence based articles and reports [13]

• Multiple survey comparison reports [9]

• Ethnicity, age and patient experience [3]

• Sociological studies [7]

### Excluded papers

Of the 23 papers excluded, five addressed the surveys' impact in general without analysing survey data, these detailed:

• Why it is important to measure patients' experiences, what aspects you should measure, and methods of collecting information [19];

• A guide to assist trust boards and stakeholders to determine the most appropriate methods of measuring patients' experience; including feedback mechanisms [20];

• The CQC's performance indicator calculation methodology for patients' experience, including categorisation, domain structure and scoring [21];

• NHS trust representatives' perceptions of the survey programme, what would encourage greater use and current obstacles against adoption [22], and

• Using the inpatient survey results, as one of many factors, when choosing a hospital for elective admission [23].

Four articles discussed the findings of other papers that analysed data but did not report independent analyses of survey data [24-27]. Four more papers made no reference to the acute trusts adult inpatient survey:

• Two discussed other surveys and were picked up by a search of 'inpatient' and 'survey' separately [28] and 'inpatient survey' (of orthopaedic patients) [29];

• Palmer [30] was identified by the Hospital Consumer Assessment of Health Plans (HCACPS) 'national inpatient survey', and

• Sheikh [31] responded to an article that analysed a Swiss 'inpatient survey'.

Shipton et al. [32], Stevens et al. [33] and Davies et al. [34] also did not use data, although authors did adopt the star ratings system as a measure of performance. Peytremann-Bridevaux et al. [35] used an adaptation of the Picker inpatient survey questionnaire for a study in University Hospital of Geneva, but did not make use of the English national inpatient survey data. A paper by Crow et al. [36] used the survey data for one trust but did not refer to national data.

Two reports discussed ethnicity in terms of response rates and increasing representation. One reported the demographic profile of responders, but not their reported experiences of care [37]. The second reviewed literature outlining strategies to increase the response rate of minority groups [38]. Likewise, Jumaa [39] referred to the response rate of the inpatient survey with no experience data or reference to year.

Finally, two articles outlined local projects that made use of trust level survey results to improve local services and measure change. The first reviewed results alongside Patient Advice and Liaison Services (PALS) [40]. The second used survey results as a measure for the success of pain improvement techniques [41].

### Annual survey reports

Annual key finding reports were identified for 2002 [14], 2004 [42], 2005 [43], 2006 [44], 2007 [45] and 2008 [46]. The Picker Institute Europe produced all of these on behalf of the regulatory body responsible for the survey at the time. In 2008, the key findings paper was accompanied by an historical comparisons document [47] and a web-based information page, which included a briefing report and trust level reports. In 2009 the historical comparisons document [48] and website [15] replaced the key findings report.

The first report in 2002 covered every question in the survey through descriptive explanations, bar charts and tables. Respondent demographic characteristics such as age, ethnicity and health status, were reported alongside Trust characteristics such as size and location. Each demographic variable was related to the questions. Older, male and white patients reported better experiences. Patients with poorer self-rated health status and those living in London reported less favourable experiences. Overall, 74 per cent rated the care they received as excellent (38 per cent) or very good (36 per cent).

In 2004, the report was reduced to descriptive national findings for key domains such as Patient Care and Treatment and Leaving Hospital. Results for each question were tabulated and also cross-tabulated to show percentage change between 2002 and 2004. All reported findings were statistically significant. Those with poorer health were more negative about the care they had received. Black, minority and ethnic (BME) groups, particularly people of South Asian origin, were more negative, though the differences were quite small relative to other factors. As in the previous survey, older people, men, elective patients, people living outside London and those who were treated in specialist Hospital Trusts reported more positive experiences. The most significant factor in explaining variations in patients' experiences was self-reported health status; those who reported their own health to be good, very good or excellent tended to report a more positive experience of care.

The 2005 report was more comprehensive than previous years. Cross-tabulated results included demographic data on respondents and non-respondents, and displayed 'all respondents' beside '18 and over' to allow for comparison to 2004. Only significant findings were included and the Bonferroni correction method applied. Due to the "differences in the sample" (16 and 17 year olds not included), results for the 2004 survey were not included in the annual reports from 2006 onwards [44].

The 2006 to 2008 surveys followed the same structure as 2005, with increasingly sophisticated analyses. Comparisons between years were tested for significance using Z-tests and the Bonferroni method. From 2008, tabulated results for the relevant years were simplified. Results were presented in full for all response options, with no further grouping or comparisons. Significant changes over time were sought by comparing the last two years (2007 and 2008) with the survey results from the first year (2002).

In 2004 selected 'free text' comments were included to support key findings. It was not until 2007 that the full free text comments were included in an additional summary report [49] which showed that nearly 60 per cent of respondents wrote at least one comment suggesting that these free text comments are also a rich source of data which have not been analysed in any other way apart from this one study conducted by the Picker Institute Europe in 2007.

### Evidence based articles and reports

A number of reports and articles identified through the review referenced the inpatient survey as supporting evidence. The papers below quoted figures from the annual survey reports:

• The Patients Association (2009) used the overall care rating to highlight poor treatment of elderly relatives [50],

• Vere-Jones (2006) quoted 20% of patients not receiving help with their food as part of "Cash-strapped trusts" saving funds through catering cut backs [51],

• Handley (2009) wrote about Walsall Hospital NHS Trust, who used discharge results to provide follow up calls to patients as part of a pilot study [52],

• Pickersgill (2010) discussed nurses influence over survey questions, such as patients' finding someone to talk to about their concerns [53],

• Staines (2008) examined the influence of the media on public perception, such as results for cleanliness following reported outbreaks of c.Difficile [54],

• Swain (2007) highlighted the benefits of working with patients; that the specific nature of survey questions provided precise guidance of where services might be going wrong [55],

• The Healthcare Commission (2006) used the survey to review the management of the admissions process for inpatients [56],

• The Healthcare Commission (2007) reported on specific questions, such as privacy and talking in front of the patient as if they were not there, to highlight "dignity in care for older people while in hospital" [57]

• Fitzpatrick et al., (2005) wrote about health and health care inequalities in England based on demographic information collected [58], and

• The NHS Confederation (2010) used the inpatient survey as a measure for targeted improvement pilots at local trusts [59].

One article solely reported on the findings of the inpatient survey for 2009 [60], highlighting differences from year to year and concludedthat while some improvements have been made, there are "still key areas of concern" such as 45 per cent of patients reporting a lack of information regarding potential side effects of new medication.

The authors of one study adapted the Picker Institute questionnaire to conduct an in-house pilot to test how a hospice was performing when compared to national hospital results for 2007 [61]. Unfortunately the sample size was too small to draw any conclusions.

Finally, an internal publication produced by the Department of Health used factor analysis to determine a model that linked patient experience to satisfaction, based on 2001/02 inpatient survey data [62]. The paper also reported analysis produced by the University of Sheffield using inpatient survey data for 2003/04, which sought to explain the variation between patients' experiences in the five core domains identified in the NHS Outcomes Framework 2011/2012 [5] as set out above. In sum, there is a substantial body of work that has made use of the national inpatient survey. The fact that so many authors have drawn on these data for diverse purposes does suggest that it is an important national resource and this should be considered in debates about the future of the survey programme.

### Multiple survey comparison reports

Nine papers made use of the national inpatient survey to both enhance an argument and to draw conclusions with other national NHS surveys. One such paper, A *High-performing NHS? *[63], reviewed the evidence on whether increased Government NHS funding had improved eight core domains from 1997 to 2010. The domains comprised access, patient safety, promoting health and managing long-term conditions, clinical effectiveness, patient experience, equity, efficiency and accountability. Key points, the situation in 1997, progress since then and future plans were discussed for each domain. The study included inpatient survey results for 2008 with healthcare surveys from different years and specialties, such as mental health. The inpatient survey results were reported briefly; patients ability to choose the hospital where they were treated had increased from 28 per cent to 33 per cent from 2007 to 2008, 21 per cent of patients reported not being given enough information about their condition or treatment, and there had been a slight increase in patients reporting that they had been asked their views on quality of care while in hospital (6 per cent to 9 per cent from 2002 to 2008). Similarly, the NHS Confederation's *Lost in translation *[64] briefly reported inpatient survey results for 2005. They concluded that patients report "high levels of satisfaction with the NHS and care they [patients] receive", with 92 per cent describing their care as good, very good or excellent (the other options being 'fair' or 'poor'). The findings were used to support the contention that patients' evaluation of their care is more positive than the predominantly negative perceptions communicated by the media and the general public.

Two reports provided a national overview of the NHS. One gave a snap shot of "patient and public expectations, experiences and evaluations" [65]. The Chartbook concluded that though some areas have improved, such as access to care, important variables such as patient engagement in decision making have not. The second report briefly discussed the inpatient survey from 2002 to 2007 as part of *An Economic Health Check *providing an overview of the NHS [66]. The report described a "largely static picture" in five key areas: access and waiting; safe, high quality, coordinated care; better information, more choice; building closer relationships and clean, friendly, comfortable place to be.

A further six reports were produced by the Healthcare Commission or Picker Institute Europe. The first report, *State of Healthcare 2007 *[67], highlighted areas that had improved as well as areas where further improvement was needed. The Picker Institute Europe sought to answer the question "*Is the NHS getting better or worse?" *in an overview of 15 national surveys between 1998 and 2004 [68]. One of the reported conclusions was that government targets and coordinated action have had a positive effect on patients' experience. A similar report examining trends of inpatients' experiences between 2002 and 2004 [69] also concluded that coordinated action facilitated improvement. A report from the Picker Institute, *Is the NHS becoming more patient centred? *[70], covered 26 surveys from 2002 to 2007, including inpatient data from 2005 and 2006 and also concluded that "many aspects of care targeted by the government have improved significantly". The report also highlighted significant improvements, such as being treated with dignity and respect, and areas of continued concern, such as cleanliness and food.

Finally, the Healthcare Commission produced a report that analysed the national patient survey programme for 2003/04 [71]. The report covered inpatient survey data from 2004 and used multiple linear regression analysis to show that self-reported health was the most significant variable for explaining variations in patients' experience. The analysis also revealed that elective patients, those admitted to specialist trusts, those admitted outside London, older people and men were likely to respond more positively which supports the conclusion that there are important socio-demographic variables that need to be taken into account when interpreting the survey results.

### Ethnicity, age and patient experience

One study investigated whether self-reported ethnic group influenced patient experiences [72] using inpatient survey data from 2006 as well as other healthcare surveys such as the outpatient survey (2004/05). The authors caution against a too literal interpretation of their findings, however, their analysis suggested that compared to the largest category, "white British", most BME groups in England were more negative about their experiences as patients, particularly in responses to questions about access and waiting times, involvement in decisions about their care and treatment, and the quality of information given to them. They were also more likely to say that staff talked about them "as if they were not there". Only those respondents who described themselves as "white Irish" were more likely to report more positive experiences than the baseline category of "white British". Ethnic differences were least marked in the inpatient survey compared to surveys from different healthcare settings, such as the community. This study was updated in 2009 [73] and once again, the results showed that apart from white Irish, BME populations were less positive about their experiences of care, particularly with regard to access and waiting and relationships with staff.

In 2001, the UK government declared its intention to "root out" age discrimination in the health service through the *National Services Framework for Older People *[74]. Lievesley et al. [75] used inpatient survey data for 2004 along with other healthcare surveys to assess whether ageism and age discrimination were still apparent in the NHS. The report revealed that the oldest (81+) and youngest (16-35) hospital patients were most likely to feel that doctors and nurses talked about them "as if they are not there". Patients aged over 81 were also less likely than those aged between 51 and 80 to rate their overall care as "excellent". The authors quoted results from the 2006 inpatient survey as described in the *National Services Framework for Older People: *older patients were particularly affected by hospital management issues, such as privacy, continence management, single sex accommodation and provision of nutritious food. A number of reports have documented that older patients tend to be less critical [57,71], making the more negative evaluation of the very oldest patients in this report all the more striking.

### Sociological studies

Several studies have attempted more complex and theoretically informed analyses using the national adult inpatient survey. These include studies that correlate other variables with overall evaluation of care, studies that compare the inpatients survey results to the NHS staff survey and one study that investigated how workforce and community characteristics affect levels of "civility" in acute Trusts.

A report by Ipsos MORI, *Frontiers of performance in the NHS II *[76], analysed inpatient survey data from 2006 along with survey data gathered from patients treated in the community from 2005. The variables correlating most strongly with "overall rating of care" were with being treated with dignity and respect, cleanliness of the room and ward, and being included in treatment decisions. The "relative importance" of these factors was calculated, with dignity and respect being the most important (59 per cent), followed by being involved in decisions (28 per cent), and then cleanliness (13 per cent). The authors used stepwise regression to identify these "most important" factors, but did not address the well-known criticisms of the stepwise approach [77]. Among these problems are that standard errors and *p*-values are biased toward zero and parameter estimates are biased away from zero. These problems are known to be especially severe in the presence of collinearity. The method by which they calculated the "percentage importance" of each factor was also not explained.

Ipsos MORI also investigated the association between inpatient satisfaction and characteristics of the geographical area in which the hospital was located. The authors based their characterisation of a trust's "local population" on the closest 2,100 output areas (as defined by the Census [78]) and found only relatively weak associations between patients' satisfaction and ethnic divisions and age, and no association with the index of multiple deprivation. They identified two external factors as the most important, again using stepwise regression. These were whether the trust was in London and the percentage of the local population aged under 16. They then compared "predicted" (using these two factors in a regression) and observed ratings of overall inpatient care.

**Figure 2:**
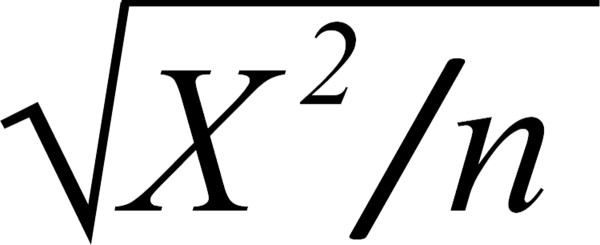
**Phi statistic**.

Doyle et al. [79] conducted a similar analysis to demonstrate a way that trusts could identify the "key drivers" of quality. The paper compared strength of association between overall satisfaction and various components by generating 3 × 2 contingency tables, with three categories of overall satisfaction, Positive, Fair, or Poor. Data from 2006 and 2007 were analysed separately. Chi-square tests were then performed on the contingency tables to test for a statistically significant association, and finally Cramer's V was calculated to assess the bivariate "strength of association" (though it is noted that since one of the variables has two categories, it is actually the phi statistic [see Figure 2]). No correction was made for the multiple pairwise comparisons that were carried out. The analysis was at the patient level, but no allowance was made for clustering within trusts. The authors report that being treated with respect and dignity was the most important component of overall satisfaction, followed by how well doctors and nurses worked together, and confidence and trust in nurses.

Sizmur et al. [80] investigated whether patients' considered certain aspects of their experience of care to be more important than others or if all were felt to be equal. The author tested which elements of the 2008 inpatient survey were most highly correlated with 'overall satisfaction' as measured by "Overall, how would you rate the care you received?" To ascertain the survey items with the strongest relationship to overall satisfaction, basic correlation analyses were applied to individual questions and overall satisfaction, at the patient level. Composite scores were created that revealed consistency and coordination of care, nurses, and patient involvement to be most strongly correlated with overall satisfaction. Multivariate stepwise regression was applied to account for confounding factors, such as gender or admission to hospital, and to identify specific factors that might predict satisfaction independently. The method resulted in similar predictors of overall satisfaction as the previous method, such as consistency and coordination of care. The author also performed analysis at the trust level, but suggested that as the variance accounted for implied high levels of multicollinearity (95 per cent), the results were "likely to be unstable".

Two papers investigated whether there was a homology between the responses on the staff and the inpatient surveys. Raleigh et al. [81] used twenty eight staff survey items as explanatory variables in regressions involving four inpatient survey responses from 2006 data. Results were aggregated to the trust level to allow the two data sets to be merged. Dummy variables for whether the trust was inside or outside London and trust type (general, teaching or specialist trust) were included in all these regressions. The selection of dependent variables was carried out using stepwise regression. The authors acknowledge some of these problems in their discussion, but do not explain why they did not use alternative, more robust methods such as LASSO or LAR [82]. They argue that their results "...show some significant patterns, indicating that the safety and quality of services and patient experience could improve if trusts acted on feedback from their staff."

Dawson (2009) asked whether the experience of staff working in the NHS was reflected in patients' experience of care [83]. Using data from 2007 to test for correlations between trust level aggregated inpatient and staff survey items he found a staggering 12,214 bivariate correlations of which 56 per cent were statistically significant. No level of significance was given, and there was no evidence of Bonferroni or alternative multiple test correction (a Bonferroni correction would imply a level of significance of 0.05/12214 ≈ .000004, and consequently fewer statistically significant correlations). Some of the key findings were intuitively plausible, for example, high levels of bullying, harassment and abuse against staff by outsiders were related to many negative patient experiences. Similarly, inpatients' perceptions of the adequacy of staffing levels and the amount of dignity and respect with which they were treated were correlated with employee's feelings of work pressure and staffing levels. However, some findings lacked any theoretical or intuitive support, such as higher numbers of staff who have had health and safety training leading to more patients perceiving staff as conscientious and available. This suggests that a stronger theoretical framework could be a useful guide to future analyses.

The Healthcare Commission's *Acute Hospital Portfolio Review: Ward Staffing *[84] also linked inpatient and staff survey data and evaluated changes in ward staffing since the Audit Commission's first investigations in 2000/01 [85]. They reported a very strong relationship between the use of temporary "bank" and agency staff, indicating high levels of vacancies and low levels of patient satisfaction. London hospitals had high vacancy rates and used temporary staff more frequently than hospitals outside the capital. This relationship was so strong that it made it difficult to investigate other staff variables related to patients' evaluations of care so separate analyses had to be conducted on Trusts inside and outside London. These analyses showed that higher proportions of registered, more experienced and skilled staff, greater numbers of staff who were satisfied with their jobs and had lower expressed intention to leave their current job were related to better patient evaluations of care. Consistent with the Audit Commission's earlier findings, the Healthcare Commission found no relationship between the total number of nurses or total expenditure and patient satisfaction. Together, these findings suggest that to improve patients' experiences, hospital trusts should invest in a richer skill mix (more experienced and skilled nurses), rather than just increasing the number of nursing staff. Although these results could be important in improving the quality of care in the NHS, it was difficult to critically appraise the research conducted by the Audit Commission as the report did not give detailed information about the data used and the ways in which they were analysed. Their report was originally backed up by a detailed document explaining the statistical analyses but a Freedom of Information (FOI) request to the CQC confirmed that this paper is no longer available.

The final article in this review used inpatient survey data to derive a trust measure of the "civility" of staff towards patients, King et al. [86]. The measure was a 14-item scale, using questions such as being treated with dignity and respect. Additional data sources were used to calculate the ethnic diversity of the medical and nursing workforce in the trust, the similarity of the ethnic makeup of the workforce and the local population, and the trust's performance. Civility was found to be negatively associated with ethnic diversity in the medical and nursing workforce. Low civility was in turn associated with poor trust performance. On the other hand, similarity of the ethnic makeup of workforce and local community was associated with increased "civility" and hence with better trust performance. The study had a strong theoretical framework from which hypotheses were derived and tested. A number of different datasets were used and data were analysed using appropriate statistics. One problem that remains is that it was not entirely clear that the 14-item scale actually measures civility and this should be tested in future research.

## Discussion

After a systematic search of the published literature, we identified a number of reports and publications that were based on analyses of national adult inpatient survey data conducted in England from 2002 to 2009. These explored the role of ethnicity, age and factors that influence patients' experience of care and assessed the association between staff and community characteristics on patients' evaluation of care.

Some reports used a single year of data and others used multiple years providing historical comparisons or trend analysis. Many of these were compiled by the organisations involved with co-ordinating or collecting the survey data. Results were mostly displayed descriptively, with more recent papers applying more advanced analytical techniques. Many of the identified papers did not perform original analyses and used reported outcomes from official annual survey reports. Sometimes the data were used in questionable ways, such as comparing different datasets as if they were alike and using patient level or trust aggregated data. The adopted methodologies varied greatly and the choice of statistical techniques was not always explained.

The question "Overall, how would you rate the care you received?" was used by many papers to gauge the importance patients' placed on different aspects of their care. However, patient surveys were emphatically not designed as satisfaction surveys. Previous research has shown that patients tend to be reluctant to make negative comments about their overall experience and satisfaction measures provide an unreliable measure of quality [87]. Asking patients to report in detail on "what happened" is much more useful. There is consequently a real need for the development of a summary score which combines all of the questions included in one survey to create a dependent variable that better represents the breadth of topics covered.

One of the findings from the national inpatient surveys was that some patient characteristics relate in a statistically significant way to their evaluation of care, for example, age, gender, ethnicity, their evaluation of their own health status and whether or not they were admitted as an emergency. Findings also report that Trusts outside of London tend to receive more positive responses than those inside the capital [71]. A recent paper suggested that ethnic diversity, both in the Trust and the local community, can also shape patients' experiences [86]. Studies linking data from the staff surveys to the inpatient survey demonstrate a relationship between the two, and ward staffing characteristics has also been shown to contribute to patients' experiences, for example, it is not solely a question of staffing volume, but of employing more experienced and skilled nurses [84].

One of the most important findings from the trend analyses is that those areas that show sustained improvement such as waiting times, cleanliness, hand-washing and mixed sex accommodation are areas which have been the focus of national campaigns, using a range of mechanisms including targets, incentives and penalties to change behaviour [55,68-70]. All too often national reports document similar findings from year to year, exaggerating small changes by marking them as statistically significant (which is a function of the large sample size rather than large changes in the percentages of responses). Overall, it seems that there was very little improvement in patients experience at the aggregate level from 2002 to 2009.

The local use of inpatient survey data for quality improvement has not been addressed here. Although the search strategy was thorough and systematic regarding national usage, it would have required a great increase in resources to fully investigate individual NHS trust publications or the grey literature, where local quality improvement studies might be found. It is known that survey data are being used locally, for example alongside PALS and steering groups to improve care records [40], to manage pain more effectively [41] or to aid local education programmes [88]. However these projects are not necessarily officially documented or nationally publicised. Investigations into local level usage and trust movement against the national average from year-to-year would be incredibly beneficial, with the potential to highlight successful schemes for national application.

The emerging picture is that the inpatient survey is not in itself a quality improvement tool. It can monitor trends and can provide comparative data but simply providing hospitals with patient feedback does not automatically have a positive effect on quality standards. The survey programme has revealed that focusing attention on specific areas or devising targets that hospitals are expected to attain, can have a beneficial effect on patients' reported experiences. The DoH address local targeting through the *Commissioning for Quality and Innovation (CQUIN) payment framework *[89,90], which incentivises "locally agreed quality improvement schemes" through proportioned conditional income. Questions from the adult inpatient survey are used as a measure in the CQUIN model.

There is an opportunity for individual academics and research teams to produce theoretically informed, methodologically sound and socially significant research using data from the national inpatient survey. As public spending is reduced in the in times of economic hardship, researchers will increasingly be encouraged to use data that are readily available, rather than collect new datasets. Funding bodies such as the National Institute for Health Services Research Standard Delivery and Organisation (NIHR/SDO) [91] are providing opportunities to support such projects, which may increase interest in the analysis of large national datasets and this survey in particular. With many countries around the world basing their research on inpatient survey questionnaires [7], there is also the potential to conduct comparative analyses using international datasets. Such collaborations would enable the transfer of knowledge and best practice to improve in many healthcare systems.

## Conclusions

The survey of adult inpatients is now well established in the NHS and is emulated in countries around the world. The principle that patients must be consulted and that their feedback is an important indicator of hospital performance is now embedded in the NHS. This review shows however that information alone does not automatically translate into improved experience of care. Sustained improvement tends to be achieved when backed by national government campaigns and targets, coupled with incentives and penalties. The survey programme shows that the NHS has tried over the last decade to move away from paternalism towards a focus on patients' experiences, but there is still some way to go. Finally, it has been shown that there is a need for further investigation into local patient-driven improvement schemes and that there is a great deal of potential for further analysis of national adult inpatient survey data.

## Abbreviations

BME: Black and minority ethnic; CHI: Commission for health improvement; CQC: Care quality commission; CQUIN: Commissioning for quality and innovation; DARE: Database of abstracts of reviews; DoH: Department of health; EPPI: Evidence for policy and practice information and co-ordinating centre; ESRC: Economic and social research council; ESDS: Economic and social data service; HCACPS: Hospital consumer assessment of health plans; MeSH: Medical subject headings; NHS: National health service; NIHR/SDO: National institute for health research service delivery and organisation; NIS: National inpatient survey; PALS: Patient advice and liaison services; PCT: Primary care trust; PICO: Population/patient, intervention/exposure, comparison, outcome; QRP: Quality and risk profiles; RCN: Royal college of nursing; UKDA: United Kingdom data archive.

## Competing interests

The authors declare that they have no competing interests.

## Authors' contributions

EW conceived the study idea and title. AD conducted preliminary literature search, designed and implemented search strategy, collected articles, drafted the manuscript and supplementary files. EW and DNB drafted the critical appraisal of papers (Patient experiences, Toward Discussion and Discussion). AD, DNB and EW revised and reviewed the manuscript. Final version reviewed and agreed by all authors.

## Pre-publication history

The pre-publication history for this paper can be accessed here:

http://www.biomedcentral.com/1472-6963/12/71/prepub

## Supplementary Material

Additional file 1 **Websites journals databases and RCN search**. Click here for file

Additional file 2 **Search terms and mechanisms**. Click here for file
