# Supplementary material for: The National Adult Inpatient Survey conducted in the English National Health Service from 2002 to 2009: how have the data been used and what do we know as a result?
Source: BMC Health Serv Res. 2012 Mar 21;12:71. doi: 10.1186/1472-6963-12-71 (PMC3355017; doi:10.1186/1472-6963-12-71)
Supplement: Additional file 1 — Websites journals databases and RCN search. [file 1472-6963-12-71-S1.PDF]

Additional file 1: websites, journals, databases and RCN search

Section 1: websites, journals and databases searched

Healthcare Commission approved organisations [12]:

- BMG Research
- CAPITA Health Service Partners (formally NHS Partners' Research and Information)
- GFK NOP
- Ipsos MORI
- Marketing Sciences
- MSB Ltd
- The National Centre for Social Research
- Patient Dynamics
- Patient Perspective
- Picker Institute Europe
- Quality Health
- SNAP surveys

Additional sites searched [7]:

- Google Scholar
- CQC
- NHS Survey
- NHS Evidence
- Department of Health
- The Health Foundation
- University of York Centre for Reviews & Dissemination

Journals and databases searched [21]:

- Academic Search Premier
- British Nursing Index
- CINAHL
- MedLine
- PubMed
- Trip Database
- BMJ Journals Collection
- Cambridge Journals
- Cochrane Library
- DOAJ
- EThOS
- Free Medical journals
- Informa world
- IngentaConnect
- Intute
- Jama & Archives Journals
- Myilibrary
- Sage Journals
- Science Direct
- Wiley Interscience
- Wounds UK

Section 2: Royal College of Nursing literature search

Note: The RCN search was conducted from 2005 to 2009 as this was prior to the study extension to 2002.

Initial request:

Subject: Acute Trusts: Adult Inpatient Survey

Data detail: Part of the National Patient Survey Programme; surveys all inpatients discharged in June, July and August of each year from 2005 to 2009.

Search detail: Papers, journals, articles that make use of these data; papers may be based on individual institutions, but must make use of the national dataset.

Including keywords: National, Adult Inpatient Survey, Patient, Acute Trust, NHS

Specific requirements:
Includes: Includes national adult inpatient survey data from 2005 to 2009, published 2005 to date
Excludes: Surveys based solely on maternity or psychiatric patients, outpatients

Search requested: 16th July 2010

Search results:

Result 1: 26th July 2010
Items found: 0
Sources: BNI and Medline; PsycINFO, AMED, EMBASE, HAPI, HMIC, Maternity & Infant Care, and Social Policy & Practice, Web of Science and Google Scholar

Result 2: 30th July 2010 (with adapted search criteria: 'Acute trusts and patient care')
Items found: 8 (1 applicable, but not unique from researchers' findings)
Sources: As above.
