# Supplementary material for: The National Adult Inpatient Survey conducted in the English National Health Service from 2002 to 2009: how have the data been used and what do we know as a result?
Source: BMC Health Serv Res. 2012 Mar 21;12:71. doi: 10.1186/1472-6963-12-71 (PMC3355017; doi:10.1186/1472-6963-12-71)
Supplement: Additional file 2 — Search terms and mechanisms. [file 1472-6963-12-71-S2.PDF]

Additional file 2: search terms and mechanisms

Section 1: search terms

All searches included variations of ‘inpatient survey’, except ‘patient survey’ as ‘patient’ was not specific enough in the areas being searched. These included ‘inpatients survey’, ‘in-patient survey’ and ‘in-patients survey’ as an exact phrase and individual words using near to or adjacent word functionality (where possible) to ensure association. The search was further refined using key words such as ‘adult’, ‘national’ and ‘acute trust’. Where definable in the search, the above would feature in the abstract or title.

Where Medical Subject Headings (MeSH) or equivalent were available variations of ‘healthcare survey’ (including: healthcare surveys; health care survey; health care surveys), ‘care survey, health’ (including: surveys), ‘survey, health care’ (including: survey healthcare; surveys, healthcare; surveys, health care) and survey (including: surveys) were used. Search criteria were adapted according to search engine capability. The search mechanisms used to identify research cited in this paper have been documented in Section 2 below.

Section 2: search mechanism

The tables below contain the search mechanisms applied to locate the research discussed in this paper; only those that gave a return have been included, the full search may be supplied on request.

Note: terms will not always include plurals as some search engines run this automatically. Where the same result is given for a single or plural, these have been presented in the same search term. Research identified has been listed against the first source it was located only.

Part a: WEBSITES [n=26]

Location/Date search: Picker Institute Europe (01/10/2010 - updated Jan 2011)

Search term	Full Articles Retrieved
Inpatient survey<br>Returned: 82 , Retrieved (unique): 2	• *Is the NHS getting better or worse? An in-depth look at the views of nearly a million patients between 1998 and 2004.* [68]<br>• *Trends in patients' experience of the NHS* [69]

Location/Date search: CQC (04-10-2010 - updated Jan 2011)

"Inpatient survey"<br>Returned: 9, Retrieved (unique): 3*	* All searches led to links; documents found through searching links<br>• *National NHS patient survey programme: Survey of adult inpatients 2008: Full national results with historical comparisons* [47]<br>• *National NHS patient survey programme survey of adult inpatients 2009: Full national results with historical comparisons* [48]<br>• *Acute hospital portfolio: Management of admission in acute hospitals* [56]
"Inpatients survey"<br>Returned: 1, Retrieved (unique): 3*	
"Inpatient" AND "survey"<br>Returned: 16, Retrieved (unique): 3*	
Inpatient/s survey<br>Returned: 10, Retrieved (unique): 3*	

Location/Date search: Department of Health (07-10-2010 - updated Jan 2011)

"Inpatient/s survey", 01/01/2002 to 01/01/2011<br>Returned: 7, Retrieved (unique): 1	• *Acute inpatient survey: national overview 2001/02* [14]
Inpatients survey (in title), 01/01/2002 to 01/01/2011<br>Returned: 263, Retrieved (unique): 2	• *Report on self reported experience of patients from black and minority ethnic groups* (2008) [72]<br>• *Report on the self reported experience of patients from black and minority ethnic groups* (2009) [73]

Location/Date search: NHS Evidence (07-10-2010 - updated Jan 2011)

Inpatient survey<br><br>Returned: 3,276, Retrieved (unique): 7 (+ 1 duplicate)	• *Patients… not numbers, people… not statistics* [50]<br>• *Caring for dignity: A national report on dignity in care for older people while in hospital* [57]<br>• *Feeling better? Improving patient experience in hospital* [59]<br>• *A high-performing NHS? A review of progress 1997–2010* [63]<br>• *Is the NHS becoming more patient-centred? Trends from the national surveys of NHS patients in England 2002-07* [70]<br>• *Ageism and age discrimination in secondary health care in the United Kingdom* [75]<br>• *Core domains for measuring inpatients' experience of care* [80]

Location/Date search: NHS Survey (07-10-2010 - updated Jan 2011)

Search term	Full Articles Retrieved
Publications>Previous Survey results>Acute Hospital Trust Surveys<br><br>Returned (using search): 691<br><br>*(Search adapted to search capacity of website)*<br><br>Retrieved (unique): 6 (+2 duplicates)	• *Patient survey report 2004: Adult Inpatients* [42]<br>• *The key findings report for the 2005 inpatients survey: The acute coordination centre for the NHS acute patient survey programme* [43]<br>• *The key findings report for the 2006 inpatients survey: The acute coordination centre for the NHS acute patient survey programme* [44]<br>• *The key findings report for the 2007 inpatients survey: The acute coordination centre for the NHS acute patient survey programme* [45]<br>• *The key findings report for the 2008 inpatients survey: Acute coordination centre for the NHS acute patient survey programme* [46]<br>• *Inpatient survey 2007: Analysis of the patients' free text comments* [49]

Location/Date search: The Health Foundation (07-10-2010 - updated Jan 2011)

"Inpatient survey", "inpatient" and "Survey"<br>Returned: 70, Retrieved (unique): 1	• *A Quality Chartbook: Patient and Public Experience in the NHS* [65]

Location/Date search: Google Scholar (07-10-2010 - updated Jan 2011)

"Inpatient survey", 2002 to 2011<br><br>Returned: 383, Retrieved (unique): 3	• *State of Healthcare 2007: Improvements and challenges in services in England and Wales* [67]<br>• *Variations in the experience of patients in England: Analysis of the Healthcare Commission's 2003/2004 national surveys of patients* [71]<br>• *Do associations between staff and inpatient feedback have the potential for improving patient experience? An analysis of surveys in NHS acute trusts in England.* [81]
Google search<br>Returned: 1, Retrieved (unique): 1	• *Lost in Translation – why are patients more satisfied with the NHS than the public?* [64]

Sites searched with no relevant articles retrieved: GFK NOP, IPSOS Mori, Marketing Sciences and Cochrane Library.
Sites searched with nil returns: BMG Research, CAPITA Health Service Partners, MSB Ltd, The National Centre for Social Research, Patient Dynamics, Patient Perspective, Quality Health and SNAP surveys.

Part b: JOURNALS AND DATABASES [n=9]

Location/Date search: EBSCO (inc. Academic Search Premier, BNI, CINAHL and Medline (01/10/2010 - updated Jan 2011)

Search term	Full Articles Retrieved
"Inpatient survey"<br>(2002 to 2011)<br><br>Returned: 25, Retrieved (unique): 3	• *Understanding what matters to patients – identifying key patients' perceptions of quality* [79]<br>• *How was your care?* [52]<br>• *Good, but could do better* [53]
Inpatient survey (2002 to 2011)Returned: 114, Retrieved (unique): 1	• *Has food fallen off the NHS agenda?* [51]

Location/Date search: PubMed (04-10-2010 - updated Jan 2011)

MH: ("Inpatients"[Mesh] AND "Health Care Surveys"[Mesh]) AND 2002/01/01:2011/01/01 [dp] Returned: 136, Retrieved (unique): 2 (+1 duplicate)	• *Patients have their say.* [54]<br>• *What do patients in NHS hospitals think about their care?* [60]

Location/Date search: IngentaConnect (07-10-2010 - updated Jan 2011)

Inpatient survey Returned: 400, Retrieved (unique): 1	• *Working in partnership with patients – Why do it and what benefits can be realised?* [55]

Location/Date search: Intute (07-10-2010 - updated Jan 2011)

Inpatients survey Returned: 1, Retrieved (unique): 1	• *Acute hospital portfolio review: Ward Staffing? Healthcare Commission* [85]

Location/Date search: Sage Journals (07-10-2010 - updated Jan 2011)

Inpatient/s survey, Jan 2002 – present Returned: 11, Retrieved (unique): 1	• *Comparing hospice and hospital* [61]

Journals and databases searched with relevant articles that had already been identified: PubMed UK and BMJ Journals Collection.
Journals and databases searched with no relevant articles retrieved: Trip Database, University of York Centre for Reviews and Dissemination (inc. DARE, NHS EED & HTA), Ethos, InformaWorld and JAMA and Archives Journals.

Part c: EXPERT COSULTATION [n=5]

- *Indications of Public Health in the English Regions-4: Ethnicity and Health* [58]
- *Understanding the Drivers of Patient Satisfaction: An Analysis of Inpatient and Outpatient National Survey Data* [62]
- *Frontiers of performance in the NHS II* [76]
- *Does the experience of staff working in the NHS link to the patient experience of care? An analysis of links between the 2007 acute trust inpatient and NHS staff surveys* [83]
- *Why organizational and community diversity matter: the emergence of incivility and organizational performance* [86]

Part d: UNIQUE PAPERS FROM SECOND READER [n=1]

- *The English national health service: an economic health check: Economics department working paper no. 717* [66]

Part e: PAPERS EXCLUDED AFTER FULL PAPER REVIEWED [n=23]

Source	Article
Care Quality Commission [2]	• *Acute and specialist patient experience methodology* [21]<br>• *Variations in the experiences of patients using the NHS services in England: Analysis of the Healthcare Commission's 2004/2005 surveys of patients* [28]
EBSCO [2]	• *Health regulator promises tough new measures to tackle poor care* [24]<br>• *Does patient experience correlate to the experience of NHS staff?* [27]
Expert Consultation [2]	• *The Point of Care: Measures of patients' experience in hospital: purpose, methods and uses* [20]

	• *The impact of leadership and quality climate on hospital performance* [32]
Google Scholar [3]	• *Conducting a Needs Assessment: Patient Education* [30]<br>• *Exploring the Relationship between Senior Management Team Culture and Hospital Performance* [34]<br>• *Review of BME coverage in the Healthcare Commission's patient survey programme* [37]
IngentaConnect [1]	• *The association between body mass index and patient's experience with inpatient care* [35]
NHS Evidence [5]	• *Do patient surveys work? The influence of a national survey programme on local quality-improvement initiatives* [22]<br>• *NHS Mutual: Engaging staff and aligning incentives to achieve higher levels of performance* [25]<br>• *The Boorman review: NHS health and well-being review – interim report* [26]<br>• *Independent sector treatment centres: A review of the quality of care* [29]<br>• *Increasing response rates amongst black and minority ethnic and seldom heard groups: A review of literature relevant to the national acute patients survey* [38]
PubMed [2]	• *Exploring how to measure patients' experience of care in hospital to improve services.* [19]<br>• *Response to an Article in the April 2005 issue of Medical Care: Perneger et al. Excluded patients "too sick to complete a questionnaire" from a mail survey* [31]
Sage Journals [1]	• *Metrics, Targets and Performance* [33]
Second Reader [5]	• *London Patient Choice Project Evaluation: A Model of patients' choices of hospital from stated and revealed preference choice data* [23]<br>• *Journeying between the Education and Hospital Zones in a collaborative action research project* [36]<br>• *What is Effective Healthcare Leadership? A Case Study of the NHS in England* [39]<br>• *Re-design of Nursing/ MD Care Record* [40]<br>• *Improving Pain Assessment to Enhance the Patient Experience in Hospital* [41]
